# Prospects of Using High-Throughput Proteomics to Underpin the Discovery of Animal Host–Nematode Interactions

**DOI:** 10.3390/pathogens10070825

**Published:** 2021-06-30

**Authors:** Tao Wang, Robin B. Gasser

**Affiliations:** Department of Veterinary Biosciences, Melbourne Veterinary School, Faculty of Veterinary and Agricultural Sciences, The University of Melbourne, Parkville, VIC 3010, Australia; robinbg@unimelb.edu.au

**Keywords:** parasitic nematode, host–parasite interactions, mass spectrometry (MS), high-throughput proteomics, LC-MS/MS

## Abstract

Parasitic nematodes impose a significant public health burden, and cause major economic losses to agriculture worldwide. Due to the widespread of anthelmintic resistance and lack of effective vaccines for most nematode species, there is an urgent need to discover novel therapeutic and vaccine targets, informed through an understanding of host–parasite interactions. Proteomics, underpinned by genomics, enables the global characterisation proteins expressed in a particular cell type, tissue and organism, and provides a key to insights at the host–parasite interface using advanced high-throughput mass spectrometry-based proteomic technologies. Here, we (i) review current mass-spectrometry-based proteomic methods, with an emphasis on a high-throughput ‘bottom-up’ approach; (ii) summarise recent progress in the proteomics of parasitic nematodes of animals, with a focus on molecules inferred to be involved in host–parasite interactions; and (iii) discuss future research directions that could enhance our knowledge and understanding of the molecular interplay between nematodes and host animals, in order to work toward new, improved methods for the treatment, diagnosis and control of nematodiases.

## 1. Introduction

Diseases caused by parasitic nematodes do not only impose a significant public health burden, but also cause major economic losses to agriculture in the subtropical and tropical countries [1]. Globally, soil-transmitted nematodes alone (e.g., *Ascaris lumbricoides*, *Ancylostoma duodenale*, *Necator americanus* and *Trichuris trichiura*) infect ~472 million people, leading to a burden of ~4 million disability-adjusted life-years (DALYs) [2]. In the livestock industry, productivity and financial losses of tens of billions of dollars per annum have been estimated as a consequence of parasitic nematode infections and diseases (nematodiases) [3]. The control of nematode infections currently relies largely on the use of a limited number of anthelmintic drugs, but there is now widespread resistance in nematodes of livestock animals to most of classes of these anthelmintics. This situation means that there is an urgent need for improved interventions, including novel drug treatments and vaccines [4,5]. While vaccines could provide a safe and sustainable solution, progress in developing highly protective anti-nematode vaccines has been limited. Barbervax^®^ and Bovilis^®^ Huskvac are two of the few examples of effective vaccines against haemonchosis and lungworm disease, respectively, which have been licensed and commercialised [6]. To some extent, limited advances in this area relate to a lack of understanding of the intricate relationships between host animals and their nematode parasites as well as host immune responses induced and evasion mechanisms employed by these parasites. Clearly, the search for new intervention targets will require detailed studies of host–parasite interactions at the molecular level [7,8].

In the past two decades, with substantial progress made in the development and adoption of nucleic acid sequencing technologies (e.g., next-generation sequencing) and associated bioinformatics (e.g., automated assembly and annotation tools/pipelines), a range of genomes and transcriptomes of parasitic nematodes have been decoded, which has significantly facilitated biological explorations of these worms at the biochemical and molecular levels [9,10]. As the building blocks of cells, proteins directly perform critical biofunctions of genes *via* enzymatic catalysis, cell signalling, signal transduction as well as protein–molecule (i.e., DNA, lipid, carbohydrate and other protein) interactions. In this postgenomic era, a key research agenda is to reveal the expression patterns and functions of nematode genes in different developmental stages and sexes, and to gain a sound understanding of critical pathways and biological processes that molecules in nematodes are involved in [11,12,13,14].

Mass spectrometry (MS)-based proteomics represents an integral postgenomic technology that can be used to characterise all proteins expressed in a particular cell type, tissue and/or organism under a defined set of conditions. The use of such technology allows biological insights *via* protein identification, quantification, localisation and the definition of post-translational modifications, as well as the elucidation of protein dynamics and functions at the host–parasite interface linked to host invasion, parasite survival, host immune evasion and immunoregulation [15,16]. The availability of draft genomes and transcriptomes for >100 species of nematodes (Parasite WormBase; 28 May 2021) now strongly underpins the expansion of mass-spectrometry-based proteomic studies of parasitic nematodes.

In the present article, (i) we review MS-based proteomic approaches available to explore parasitic worms, with an emphasis on the high-throughput ‘bottom-up’ approach; (ii) we appraise recent advances that have been made in the study of the proteomes of parasitic nematodes of animals, highlighting proteins inferred to be involved in host–parasite interactions, and (iii) we provide a perspective on areas of future work that should assist in gaining an enhanced understanding of the molecular interplay of nematodes with their hosts.

## 2. Mass-Spectrometry-Based Proteomics Approaches Applied to Study Parasitic Nematodes

There are two types of MS-based proteomic strategies: ‘top-down’ and ‘bottom-up’. The ‘top-down’ approach refers to the direct measurement of intact proteins, enabling the characterisation of proteins without the need of an annotated reference genome [17]. Although it provides advantages for the definition of post-translational modifications, the ‘top-down’ approach is considered to be less suited for the high-throughput characterisation of complex samples due to technological challenges associated with fractionation and ionisation of proteins and fragmentation in the gas phase [17,18]. On the other hand, the ‘bottom-up’ approach is based on the analysis of small peptides obtained following extensive proteolytic digestion, and allows the large-scale characterisation of complex mixtures of molecules derived from whole worms, organ systems, tissues or cells. This approach allows the analysis of thousands of proteins, including those of low abundance, in a single run within a short time-frame (1–2 h) (reviewed in [17]). Advances in MS technologies and the availability of annotated nuclear genomes have meant that the proteomic investigation of proteins in complex samples (i.e., whole nematodes or tissues thereof) has shifted from ‘top-down’ to ‘bottom-up’.

Three main workflows are used for ‘bottom-up’ proteomics, applicable depending on sample complexity and the objectives of the analysis:

(i) *‘Targeted’ proteomics*: Here, selected/multiple reaction monitoring (SRM/MRM) and parallel reaction monitoring (PRM) are two commonly used methods [18,19]. Using triple-quadrupole, quadrupole-ion trap or quadrupole-time-of-flight (TOF) instruments, SRM/MRM methods allow precursor ions with expected mass to pass through a first phase of MS (MS1), after which multiple fragmented ions from the selected precursor ions are screened in a second phase of MS analysis (MS2) for target detection. In MS1 and MS2, spectrum scans are separated by a defined mass-to-charge (*m*/*z*) value. SRM/MRM enables rapid profiling of pre-determined and known subsets of proteins of interest with high specificity and quantitative accuracy, regardless of different treatments or conditions of samples [18,19]. PRM is an ion monitoring technique performed using a quadrupole-Orbitrap mass spectrometer. The principle of this technique is comparable to SRM/MRM, but assay development is more convenient. Unlike SRM, which performs one transition at a time, PRM performs a full scan of each transition by a precursor ion (i.e., parallel monitoring of all fragments from a precursor ion). Thus, PRM can be applied to analyse complex samples to achieve absolute quantification of multiple proteins to an attomole-level [18]. Nevertheless, SRM/MRM and PRM methods are less suited for discovery-based proteomics.
(ii) *Data-independent acquisition (DIA)*: This method allows the analysis of all peptides within a defined *m/z*-range to be fragmented and analysed in MS2. The aim of this method is to obtain comprehensive fragmentation mapping of proteins in highly complex samples without being limited to profiling pre-determined peptides of interest. However, there is a technical limitation in the coverage of precursor ions using the DIA approach. Consequently, it is common practice to generate ‘pseudo’ fragment ion mass spectra prior to database searching
(iii) *Shotgun proteomics*: This method [20] resembles the shotgun DNA-sequencing approach used in the genomics field. Here, proteins are digested enzymatically and subjected to liquid chromatography tandem MS (LC-MS/MS) in a data-dependent acquisition (DDA) mode. Peptides are identified by matching the mass spectra of peptide fragments with theoretical mass spectra calculated for proteins of an organism represented in an annotated genome available via a public database. Shotgun proteomics is now the ‘mainstream’ approach used for proteomic studies of parasitic nematodes. The latest applications of high-throughput proteomics to parasitic nematodes of animals are summarised in the following section. We have elected to include studies published in the last five years, because this is when most progress has been made using high-throughput proteomics due the availability of informative nuclear genomic data sets for selected socioeconomically important parasitic nematodes of animals.

## 3. Recent High-Throughput Proteomic Explorations of Parasitic Nematodes

Critical for the establishment and survival of a parasitic nematode (parasitism) in its host are the processes of host invasion, migration, feeding, reproduction and the establishment of a ‘balanced’ interaction with the host animal [21]. Such ‘cross-talk’ begins when the worm invades the host and is then maintained, relatively consistently, during the phase of parasitism. It is believed that not only does the nematode suppress host immune responses via the release of parasite-derived immunomodulators (such as secretory proteins, products and vesicles), but the host can also regulate the synthesis of proteins and other molecules in the worm [21,22,23,24].

### 3.1. The Secretome

Excretory-secretory (ES) proteins, including those actively released from the worm gut or the cuticular surface of nematode, can play key roles at the parasite–host interface. An *in silico*-prediction study [25] showed that the full complement of ES proteins (called ‘secretome’) represents a considerable proportion (~8%) of the nematode proteome. Many ES proteins represent essential molecules, such as hormones, proteases and antimicrobial peptides, which indicates that this molecular subset could present suitable targets for drugs or vaccines [26,27]. Hence, recent high-throughput proteomic studies mainly focused on comprehensive analyses of ES proteins (Table 1). Typically, ES proteins of parasitic nematodes are collected from *in vitro*-cultured parasites and then concentrated prior to LC-MS/MS analysis. Intriguingly, many of the ES proteins (described in the following) identified/characterised are not only abundant in numerous nematode species from distinct taxonomic groups, but also seem to be regulated in a stage-specific manner. They are suggested to be involved in facilitating host invasion, migration as well as nutrient acquisition, antibacterial or antioxidant activities and/or immune evasion or suppression, enabling nematode survival.

**Hookworms and related worms.** Hookworms (i.e., *Necator americanus*, *Ancylostoma duodenale* and *Ancylostoma ceylanicum*; family Ancylostomatidae) infect about half a billion people worldwide and are a significant threat to human health, particularly in underprivileged communities [28]. The secretomes of hookworm species are relatively well characterised compared with other parasitic nematodes. Initial studies of selected hookworm ES proteins began in the 1990s [29] and, subsequently, an emphasis was placed on the global characterisation of hookworm secretomes [30]. This led to the identification a series of potential anti-hookworm vaccine targets, such as sperm-coating proteins/Tpx-1/Ag5/PR-1/Sc7 (SCP/TAPS) and calreticulin [30].

Advances in high-throughput proteomic techniques and the accessibility of hookworm genomes have allowed significant progress in studying hookworm–host interactions. A particular example is the recent study of the dog hookworm *Ancylostoma caninum*. Using advanced LC-MS/MS combined with in-gel and off-gel electrophoresis, in 2017, Morante et al. [31] identified nearly three times (*n* = 315) the number of proteins reported previously (*n* = 105) by Muvenna et al. [32] in 2009. Facilitated by state-of-the-art MS and underpinned by genomic information, a re-analysis of the proteome only required a quarter of previously required amount of ES products (equating to 150 μg of protein *versus* 660 μg previously) [32]. Importantly, a number of ‘novel’ proteins (e.g., cysteine proteases ANCCAN_06649, ANCCAN_06644, ANCCAN_30567, ANCCAN_6619 and ANCCAN 06647) with immunogenic potential were reported for the first time.

Most previous studies of hookworm secretomes were conducted using *Nippostrongylus brasiliensis*, *Ancylostoma caninum* and *Ancylostoma celyanicum*, which can readily be maintained in animal models [33], facilitating the production of parasite materials for analyses. However, it was not until 2020 that the first comprehensive proteomic analysis of a secretome of a human hookworm was reported [34]. In this study of *Necator americanus* [34], a total of 198 ES proteins were identified in the adult stage, 90% of which had homologs in the secretomes of three other strongylid nematodes (i.e., *Ancylostoma caninum*, *Heligmosomoides polygyrus/bakeri* and *Nippostrongyus brasiliensis*) [31,35,36], confirming the value of these reference species for such studies. Of the 198 ES proteins identified/characterised for adult *Necator americanus*, ~ 30% of them belong to the group of SCP/TAPS proteins with one or two ‘SCP’ domains [34]. SCP/TAPS proteins are cysteine-rich secretory proteins, and are commonly detected in the secretomes of parasitic nematodes, particularly hookworms [37]. These proteins are believed to play key roles during host invasion, the penetration of tissues (e.g., skin, mucosa and connective tissues) and the transition from the free-living to parasitic stages for many nematode species [38], although the exact functions of many members of this family remain to be established. SCP/TAPS proteins have been shown to be abundant in the proteomes of a number of nematode species within clades IV and V, such as *Ancylostoma caninum*, *Teladorsagia circumcincta* and *Strongyloides ratti* [34,38]. Notably, a recent study of the secretome of *Heligmosomoides polygyrus/bakeri* (family Trichostrongylida) showed that interactions between the sexes of this species influence the composition and abundance SCP/TAPS proteins. Specifically, SCP/TAPS proteins collected from mixed-sex cultures of fourth-stage larvae (L4s) of *Heligmosomoides polygyrus/bakeri* were more abundant than those harvested from either females or males [39].

***Haemonchus contortus*.** Taking advantage of recently-updated genomic resources [40], Wang and co-workers [41] characterised ES proteins representing four distinct developmental stages or sexes (i.e., L3, L4, adult male and adult female) of *Haemonchus contortus*—the barber’s pole worm of ruminants. A total of 878 unique ES proteins were identified and quantified. ES proteins, such as proteases, SCP/TAPS proteins, glycoside hydrolases, C-type lectins and transthyretin-like proteins, were not only found to be abundantly secreted in one or more developmental stages of *Haemonchus contortus*, but there were marked differences in abundance between or among stages, indicating that some of these molecules (particularly proteases and glycoside hydrolases) are involved in nutrient digestion and acquisition as well as parasite-host interactions during the transition from the free-living to the parasitic phase [41]. In particular, the abundance of glycoside hydrolases (*n* = 13) in the secretomes of parasitic stages (i.e., L4 and adults) [41] is of particular interest, because, based on an earlier study [42], such hydrolases are indispensable molecules for energy metabolism in gut-dwelling parasites, such as *Ascaris suum*, although they have not been previously identified as abundant in parasitic nematodes that ingest blood as their main diet. This discovery, revealed using an advanced high-throughput proteomic approach, appears to have shed light on an alternative energy supply pathway in *Haemonchus contortus*, which takes place via the degradation of complex sugars.

Considerable effort has also gone into identifying and evaluating immunogenic proteins present in the secretome of *Haemonchus contortus* [43,44,45]. Using co-immunoprecipitation, combined with shotgun LC-MS/MS, Lu et al. [45] identified 114 ES proteins that interact with host T cells, including a series of proteases, SCP/TAPS proteins and C-type lectins. Thus, this study provided a first insight into the interplay between this parasite (*via* ES products) and the immune system (*via* effector cells), and also proposed possible vaccine candidates, such as the α/β-hydrolase domain protein (HcABHD), of which a recombinant version has been reported to achieve up to 70% protection against *Haemonchus contortus* challenge infection in a subsequent study [44].

***Teladorsagia circumcincta.*** Proteomics has also been carried out on *Teladorsagia circumcincta*—the brown stomach worm of small ruminants—to characterise tissue-specific ES proteins. Using LC-MS/MS coupled with comparative two-dimensional gel electrophoresis, Price and colleagues [46] identified 17 differentially released proteins, 14 of which were enriched in the secretome of mucosal-dwelling L4s of *Teladorsagia* *circumcincta*, and three of which were identified in lumen-dwelling larvae. Of the latter three proteins, peroxiredoxin was proposed to play an antioxidant role in helping mucosal-dwelling worms scavenge and eliminate host-derived reactive oxygen species.

***Angiostrongylus cantonensis.*** A proteomic analysis of ES products from young adults of *Angiostrongylus cantonensis* using LC-MS/MS [47] identified > 250 proteins, of which disulphide isomerase and calreticulin were abundantly represented. Western blot analysis inferred a disulphide isomerase, an aspartic protease, an annexin and five presently unknown proteins that induced an antibody response in infected mice [47].

***Trichuris*species.***Trichurismuris* (of mouse) and *Trichuris* *suis* (of swine) have been extensively used in animal models to study trichuriasis. Recent studies of proteins secreted from *Trichuris muris* employing LC-MS/MS have provided insight into aspects of host-whipworm interactions. For instance, Eichenberger et al. [48] identified 148 secreted proteins in adult *Trichuris* *muris*, including several protease inhibitors, such as secretory leukocyte proteinase inhibitor (SLPI)-like proteins, which play key roles in immunomodulation and wound healing [49]. In a study of *Trichuris* *suis*, Leroux et al. [50] identified 328 ES proteins, many homologs of which have been reported for other nematodes, including *Trichuris muris*, *Ascaris suum* and *Haemonchus contortus*, and are purported to be involved in inducing or modulating host immune responses.

***Trichinella*species.** Immunoproteomic studies of *Trichinella spiralis*, *Trichinella pseudospiralis* and *Trichinella britovi* have focused on ES proteins [51,52,53,54,55], with the intention of improving the immuno- or sero-diagnosis of trichinellosis. Numerous immunogenic proteins, including serine protease, enolase and peptidase inhibitors, were recognised by antibodies in the sera from patients or from experimental animals. These findings might provide guidance on selecting antigens for early diagnosis and possible vaccine candidates.

There has been an increased attention on proteases secreted by *Trichinella*. It is believed that such proteins assist the parasite during the invasion and tissue migration phases, and modulate immune responses at the host–parasite interface in multiple ways [56,57]. Using zymography, combined with shotgun LC-MS/MS, 29 proteins inferred to be proteases were identified in the secretome of infective larvae (L1s) of *Trichinella spiralis* taken from the host intestine [58]. In this species, the transcription of selected genes encoding two serine proteases—a cathepsin B-like cysteine proteinase and a zinc metalloproteinase—in the larval stage was shown by qPCR to be markedly higher than in muscle larvae. Based on these findings, it was proposed that the proteolytic enzymes directly exposed to the host intestinal milieu may mediate the worm’s penetration of the enteral epithelium and assist in immune evasion during parasite invasion and migration in the host animal.

***Strongyloides venezuelensis*.** This intestinal nematode of rodents is commonly used as a laboratory model to study the biology of *Strongyloides* more generally. Using LC-MS/MS, Maeda et al. [59] characterised 196 and 436 proteins secreted from respective adult female and infective larvae (L3s) of *Strongyloides venezuelensis*. The proteins most abundantly secreted from adult worms were those associated with glycolysis and DNA binding, whereas abundant molecules released from the L3 stage were linked to peptidase activity, embryo development and the oxidation-reduction process. Of note, a comparative analysis of protein and gene expression levels linked to secreted proteins in samples collected before and after *in vitro*-culture showed that secreted proteins were already expressed and accumulated in ‘pre-infection’ L3s, so that the proteins required are ready to be released as soon as the worm infects the host animal. These findings provide some support for the key role of ES proteins during host invasion.

### 3.2. Extracellular Vesicles (EVs)

A growing number of studies has shown that, in addition to soluble molecules (e.g., proteins, lipids and glycans), ES products of parasitic nematodes contain other parasite-derived extracellular vesicles (EVs). EVs, ranging in size from 40 nm to 150 nm, are tiny membrane-bound vesicles shed by most cells, which relate to a previously-overlooked role in delivering molecules to other cells. Based on the size of such vesicles, at least two types of EVs from parasitic nematodes have been described, namely exosomes (or “120 k”) and microvesicles (or “15 k”) [24,60]. EVs released by parasitic nematodes contain particular nucleotides (e.g., microRNAs), proteins (e.g., enzymes and toxins) and/or signalling lipids, and are likely involved in modulating innate and/or adaptive immune responses [61].

The active release of EVs from parasitic nematodes has been witnessed for key nematode species, including *Ascaris* *suum* [62], *Brugia malayi* [63,64], *Heligmosomoides polygyrus* [65], *Nippostrongylus brasiliensis* [66], *Teladorsagia circumcincta* [67], *Trichinella spiralis* [68], *Trichuris suis* [69,70] and *Trichuris muris* [48,71,72]. Given the important roles proposed for EVs at the host–nematode interface, high-throughput proteomics has been exceptionally useful to characterise the protein composition of EVs from mainly adult stages of these parasites. It is important to consider, however, that this is an emerging field of study and, as yet, the number of proteins identified in different studies of EVs varies considerably [60]. For instance, a marked difference in the number of EVs proteins (73 *versus* 364) identified in the whipworm *Trichuris muris* was reported in two independent studies [48,72]. It was suggested that the contrasting findings might be the result of the use of distinct methods for the isolation and MS analysis of EVs [60].

Although most EV proteins identified in parasitic nematodes had been reported in previous ES proteomics studies, including vaccine candidates—such as SCP/TAPS proteins and metallopeptidases—EVs proteins in different nematode species tend to display diverse profiles. An analysis of EVs in proteomic datasets of parasitic nematodes showed that, except for a few eukaryotic EVs markers (e.g., heat shock proteins and 14-3-3 protein), only five EVs proteins were commonly observed in most of the nematode species studied: two M13 metallopeptidases, one actin, one transthyretin-like family proteins and one aspartic protease [60]. As mentioned, there is still a lack of understanding of the protein composition and amounts in nematode EVs. More in-depth proteomics and investigations are needed to gain valuable insights into nematode-specific EVs proteins.

### 3.3. The Somatic Proteome

Recently, significant progress has been made in the identification of somatic biomarkers or therapeutic targets in nematodes, employing proteomics and immunoproteomic approaches. Nevertheless, most investigations have focused on studying one or two developmental stages [54,73,74,75,76,77,78], and global investigations of developmental proteomes have only recently been tackled using high-throughput methods. Since the first systematic investigation of the somatic proteome of free-living and parasite stages of the human filarioid nematode, *Brugia malayi*, approximately a decade ago [23], there have been only two studies of other filarioids—*Onchocerca volvulus* and *Onchocerca ochengi* [79,80], one of *T. spiralis* (trichina) [81] and another of *Haemonchus contortus* [22].

For *Onchocerca ochengi*, which is a model for *Onchocerca volvulus* (causing river blindness in people), Armstrong et al. [77] comprehensively characterised a total of 4260 unique proteins from multiple developmental stages (i.e., infective larvae, intrauterine microfilariae and adult worms) and fluid from intradermal nodules. Enzymes involved in redox and/or detoxification functions were widely distributed in all of the stages/samples studied, indicating that the parasite likely scavenges host-reactive oxygen and nitrogen species. The results also revealed an abundance of mitochondrial-related proteins in both vector-derived infective (L3) larvae and intrauterine microfilariae, highlighting that early larval stages of this species rely heavily on mitochondrial function for aerobic respiration. On the other hand, for *Onchocerca volvulus*, Bennuru et al. [80] reported a somatic proteome representing larval stages (i.e., L1, L2 and infective L3) derived from the black fly vector *Simulium damnosum* and ensuing larval and adult stages from the human host, which contained 7000 proteins. Overall, the global developmental proteome of *Onchocerca volvulus* was relatively consistent, in terms of protein composition, with that of the congener *Onchocerca ochengi*, but more distinct from that of the related species *Brugia malayi* [80].

In addition to these studies of filarioid nematodes, somatic proteomes have also been characterised for three different life stages (‘newborn’ larvae, ‘muscle’ larvae and adults) of *Trichinella spiralis* [81], and identified proteins expressed in a stage-specific manner. Overall, 1067 of 4691 (>20%) proteins of *Trichinella spiralis* identified were differentially expressed among the three developmental stages studied. Many of these proteins appear to be regulated to meet the physiological and biochemical requirements of *Trichinella spiralis* at particular stages of development. For instance, significantly higher levels of antioxidant-related molecules (e.g., thioredoxin peroxidases, peroxiredoxins and glutathione peroxidases) were identified in the adult stage of *Trichinella spiralis* compared with both newborn larvae and muscle larvae, indicating marked oxidative stress in this stage within the gut environment [81].

During the life-cycle of most parasitic nematodes, the initial larval stages grow substantially as they develop to the adult stage in a relatively short time period. In the case of *Haemonchus contortus*, it only takes about one month for the infective larvae (~0.5 mm long) to grow into adult worms of ~1.5–3.0 cm in length [82]. Thus, at molecular level, a strictly regulated nutritional metabolism pattern is expected, so that the high growth, cellular differentiation and developmental needs are met in the transition from the free-living to the parasitic phase of the life cycle. To gain molecular insight into the developmental biology of *Haemonchus contortus*, a total of 2487 proteins were identified and semi-quantified for five different developmental stages or sexes (i.e., eggs, L3s, L4s, and female and male adults) [22]. Bioinformatic analysis inferred that proteins involved in the metabolism of carbohydrates, amino acids and lipids or fatty acids were significantly upregulated, reflecting an increased demand for energy during the fast growth phase from the free-living to parasitic stages, similar to a previous transcriptional analysis of *Haemonchus contortus* [83,84], indicating that nutritional metabolism is under tight post-transcriptional control.

## 4. Conclusions and Opportunities for Future Research

Proteomic studies of ES, EV and somatic proteins of parasitic nematodes can underpin molecular investigations of host–parasite interactions to enable the identification of intervention targets and diagnostic markers or biomarkers. However, there is still a long way to go to achieve a comprehensive appreciation of the complexities involved in these interactions and to apply this fundamental knowledge to achieve applied outcomes, such as the development of new interventions to combat nematode infections or nematodiases. A complementary multi-omics approach [13] is likely required to achieve a paradigm shift. Here, we discuss some possibilities for future research using high-throughput proteomic methods.

First, it will be important to study the dynamic changes in protein profiles in single cells of parasitic nematodes. Fortunately, both antibody-based and isobaric carrier-based single-cell proteomic approaches have been successfully applied to mammalian cells [85,86], and we expect these methods to be applicable to invertebrate cells very soon. Although considerable work still needs to be done to optimize methods for the isolation and extraction of intact single cells from different nematode tissues, the prospects for proteomic analyses of single cells should allow profound insight into, and understanding of, nematode biology, which will better inform us about the mechanisms and pathways involved in host–nematode interactions.

Secondly, although most previous proteomics studies were focused on characterising ES and EV proteins, the molecules on the cuticle of worms cannot be ignored. Nematodes moult multiple times in their life cycle, replacing their existing cuticle with a fresh one each time. Consequently, a particular nematode species might display or express different proteins on the cuticular surface at different stages, as part of their immune evasion arsenal. For example, the attachment of host antibodies and leukocytes to the cuticle of *Toxocara* larvae stimulates rapid shedding of the surface coat, leaving behind an abandoned glycocalyx to which host immune cells remain attached [87]. Although surface protein-targeted proteomics has been conducted, to some extent, for nematode species such as *Ascaris suum* [88] and *Strongyloides stercoralis* [89], proteomic characterisation of cuticle surface-associated proteins in nematode has not drawn much attention. With the rapid development of MS imaging and the associated laser-capture microdissection approaches, the characterisation of tissue proteins in a high-throughput and spatial manner has become possible [90]. Future work should focus on utilising these modern approaches to unravel the composition and to quantify surface proteins exposed on the cuticle of nematodes, which could potentially disclose numerous biomarkers and therapeutic targets.

Another area worthy of pursuit is the study of post-translational modifications in parasitic nematodes using proteomics (mainly phosphoproteome and glycoproteome). Typically, these modification events often reversibly regulate the biochemical and physiological functions of associated proteins (e.g., phosphorylation *versus* dephosphorylation, and glycosylation *versus* deglycosylation), and also govern, to some extent, the cellular localisation of proteins [91]. Although there is currently scant knowledge of protein phosphorylation and glycosylation in parasitic nematodes, this is an emerging field in parasitology. To elucidate the roles of post-translational modifications in the host–nematode interaction, first, the occurrence of the various modification events in distinct developmental stages of nematodes should be explored in detail on a proteome-wide scale. Recently, two studies explored the phosphoproteome and N-glycoproteome of *Haemonchus contortus*, and shed the first light on some post-translational modifications in this parasitic nematode [92,93]. We hope that these advances in phosphoproteomics and glycoproteomics will stimulate similar investigations of other nematode species and will complement multiomic data sets to better understand the molecular aspects of host–nematode interactions.

## Figures and Tables

**Table 1:** Summary of recent high-throughput proteomic studies of excretory-secretory proteins from species of parasitic nematodes.

Nematode Species	Developmental Stages	Analytical Approach	No. of Proteins Identified	Year; Reference
*Ancylostoma caninum*	Adult worms (both sexes)	Proteomics; LC-MS/MS	315	2017; [31]
*Heligmosomoides polygyrus*	L4s (mixed and separate sexes)	Proteomics; LC-MS/MS	258	2021; [39]
*Necator americanus*	Adult worms (both sexes)	Proteomics; LC-MS/MS	198	2020; [34]
*Haemonchus contortus*	L3s, L4s and adults (separate sexes)	Proteomics; LC-MS/MS	878	2019; [41]
	Adult worms (both sexes)	Immunoproteomics; LC-MS/MS	114	2020; [45]
*Trichuris muris*	Adult worms (both sexes)	Proteomics; LC-MS/MS	148	2018; [48]
*Trichuris suis*	L4s and adults (both sexes)	Proteomics; LC-MS/MS	354	2018; [50]
*Teladorsagia circumcincta*	L4s	Proteomics; LC-MS/MS	17	2019; [46]
*Trichinella spiralis*	Muscle larvae	Immunoproteomics; LC-MS/MS	17	2020; [53]
	Intestinal infective larvae	Immunoproteomics; LC-MS/MS	30	2021; [58]
	Adult worms (both sexes)	Proteomics; LC-MS/MS	10	2016; [54]
*Trichinella britovi*	‘Muscle’ larvae	Immunoproteomics; LC-MS/MS	18	2020; [53]
*Trichinella pseudospiralis*	‘Newborn’ larvae and adults	Immunoproteomics; MALDI-TOF/TOF-MS/MS	28	2017; [52]
	Muscle larvae (three isolates)	Proteomics; LC-MS/MS	2591	2020; [51]
*Strongyloides venezuelensis*	L3s and female adults	Proteomics; LC-MS/MS	436	2019; [59]
*Angiostrongylus cantonensis*	Adult worms (both sexes)	Proteomics; LC-MS/MS	254	2019; [47]

Abbreviations: L3s, the third-stage larvae, L4s, the fourth-stage larvae; MALDI, matrix-assisted laser desorption/ionisation; LC, liquid chromatography; MS, mass spectrometry; TOF, time-of-flight.

## Data Availability

Not applicable.
